# Improved photovoltaic performance of silicon nanowire/organic hybrid solar cells by incorporating silver nanoparticles

**DOI:** 10.1186/1556-276X-8-88

**Published:** 2013-02-18

**Authors:** Kong Liu, Shengchun Qu, Xinhui Zhang, Furui Tan, Zhanguo Wang

**Affiliations:** 1 Key Laboratory of Semiconductor Materials Science, Institute of Semiconductor, Chinese Academy of Sciences, 100083, Beijing, People's Republic of China; 2 State Key Laboratory of Superlattices and Microstructures, Institute of Semiconductor, Chinese Academy of Sciences, 100083, Beijing, People's Republic of China

**Keywords:** Silicon nanowire, Silver nanoparticle, Surface plasmon resonance, Hybrid solar cell

## Abstract

Silicon nanowire (SiNW) arrays show an excellent light-trapping characteristic and high mobility for carriers. Surface plasmon resonance of silver nanoparticles (AgNPs) can be used to increase light scattering and absorption in solar cells. We fabricated a new kind of SiNW/organic hybrid solar cell by introducing AgNPs. Reflection spectra confirm the improved light scattering of AgNP-decorated SiNW arrays. A double-junction tandem structure was designed to manufacture our hybrid cells. Both short-circuit current and external quantum efficiency measurements show an enhancement in optical absorption of organic layer, especially at lower wavelengths.

## Background

Organic solar cells have emerged as potential energy conversion devices for several advantages, including flexibility, lightweight, semi-transparent characteristics, and ability to large-scale production at low temperature [1-3]. However, their reported efficiencies are still very low even for laboratory cells. The most crucial problems many of these devices face are limited mobility of charge carriers and rapid recombination. To mitigate these problems, some special methods, such as reducing the thickness of the active layer of solar cell and incorporating inorganic materials with high carrier mobility, have been taken for effective charge separation [4-6].

One of these inorganic materials is silicon nanowires (SiNWs) [7-9]. Most recently, some research groups have demonstrated fabrication of SiNW/organic hybrid solar cells [10-16]. These SiNWs can offer at least three advantages for solar energy conversion. First, they provide high-mobility pathway from the active interface to the electrodes for carriers. Second, they can significantly reduce reflection and induce strong light trapping between nanowires, resulting in strong absorption. Finally, they increase the contact area between the two materials.

On the other hand, application of AgNPs in organic photovoltaic devices is of considerable interest [17]. Surface plasmon resonance in AgNPs offers a promising way to enhance the power conversion efficiency (PCE) of organic solar cells as it exhibits strong local field enhancement around the AgNPs, which can increase light scattering and absorption in the organic film [18-21]. In recent years, a simple method for depositing AgNPs on silicon wafers by galvanic displacement has received renewed interests [22]. As a versatile fabrication method, it is well suited to yield films with high purity and substrate adhesion [23]. Thus, it is expected that the integration of AgNP-decorated SiNW array and polymer could lead to a simple process and high-performance solar cells.

In this work, we report an efficient approach for enhancing the PCE of SiNW/poly(3-hexylthiophene) (P3HT):[6]-phenyl-C61-butyric acid methyl ester (PCBM) hybrid solar cells by decorating AgNPs on the SiNW surface. In order to evaluate the performance of the scattering effect of AgNPs, we have prepared different diameters of AgNP-decorated SiNW array samples by varying Ag deposition duration, with a Ag-free SiNW array sample as reference. Some hybrid solar cells with the structure of Al/n-type SiNW/AgNP/P3HT:PCBM/poly(3,4-ethylene-dioxythiophene):poly-styrenesulfonate (PEDOT:PSS)/indium tin oxide (ITO) were fabricated.

## Methods

N-type silicon wafers with a thickness of 200 μm and a resistivity of 1 to 10 Ω cm were used. Vertically aligned SiNW arrays were prepared by metal-assisted chemical etching [24,25]. Silicon pieces were first immersed into an aqueous solution of 5 M hydrofluoric (HF) acid and 0.02 M silver nitrate (AgNO_3_) for 60 s at room temperature to deposit Ag particles. Then, the Ag particle-coated silicon wafers were moved into an etching solution contained in a reactive vessel for 3 min. The etching solution was made of 5 M HF acid and 0.2 M hydrogen peroxide (H_2_O_2_). When the etching processes were over, the silicon strips were dipped into an aqueous solution of nitric acid (HNO_3_) and then rinsed with deionized water to remove any residual silver. After that, the synthesized SiNW array samples were immersed in a plating solution containing HF acid (5 M) and AgNO_3_ (0.02 M) to deposit AgNPs on SiNWs. The diameter of AgNPs was adjusted by changing deposition times. For comparison, another sample without AgNPs was also prepared. In order to obtain standard spherical particles and decrease defects on the surface, the AgNP-decorated SiNW array was annealed in N_2_ at 200°C for 90 min before cell fabrication.

Before polymer coating, aluminum (Al) had been attached onto the rear side by thermal evaporation to obtain an ohmic contact. The polymer, P3HT:PCBM (refers to [60]PCBM) with a weight ratio of 1:1, was deposited onto SiNWs by spin coating (2,000 rpm, 1 min), and PEDOT:PSS was deposited onto ITO/glass substrate by spin coating (4,000 rpm, 1 min) in air. Then, PEDOT:PSS/ITO/glass substrate were coated on the P3HT:PCBM and fixed with a clip to complete the hybrid solar cell fabrication. After that, the whole substrates were baked at 110°C in nitrogen for 20 min. A hybrid solar cell without AgNPs decorated was also prepared as a reference device. The active area of all the cells was 16 mm^2^.

The morphology of SiNWs and AgNPs was characterized using a scanning electron microscope (SEM; JSM-7401F, JEOL Ltd., Akishima-shi, Japan). The crystal structures of the AgNPs were characterized by X-ray diffraction (XRD) using the copper Kα radiation. The reflection spectra were obtained at room temperature using a fiber-optic spectrometer (AvaSpec-2048, Avantes BV, Apeldoorn, The Netherlands) equipped with an integrating sphere. Current density-voltage (*J*-*V*) characteristics were measured with assistance of AM 1.5 illumination (100 mW/cm^2^). The quantum efficiency testing was performed on a DH1720A-1 250-W bromine tungsten arc source (DaHua Electronic, Beijing, China) and a Digikrom DK240 monochromator (Spectral Products, Putnam, CT, USA).

## Results and discussion

**Figure 1:**
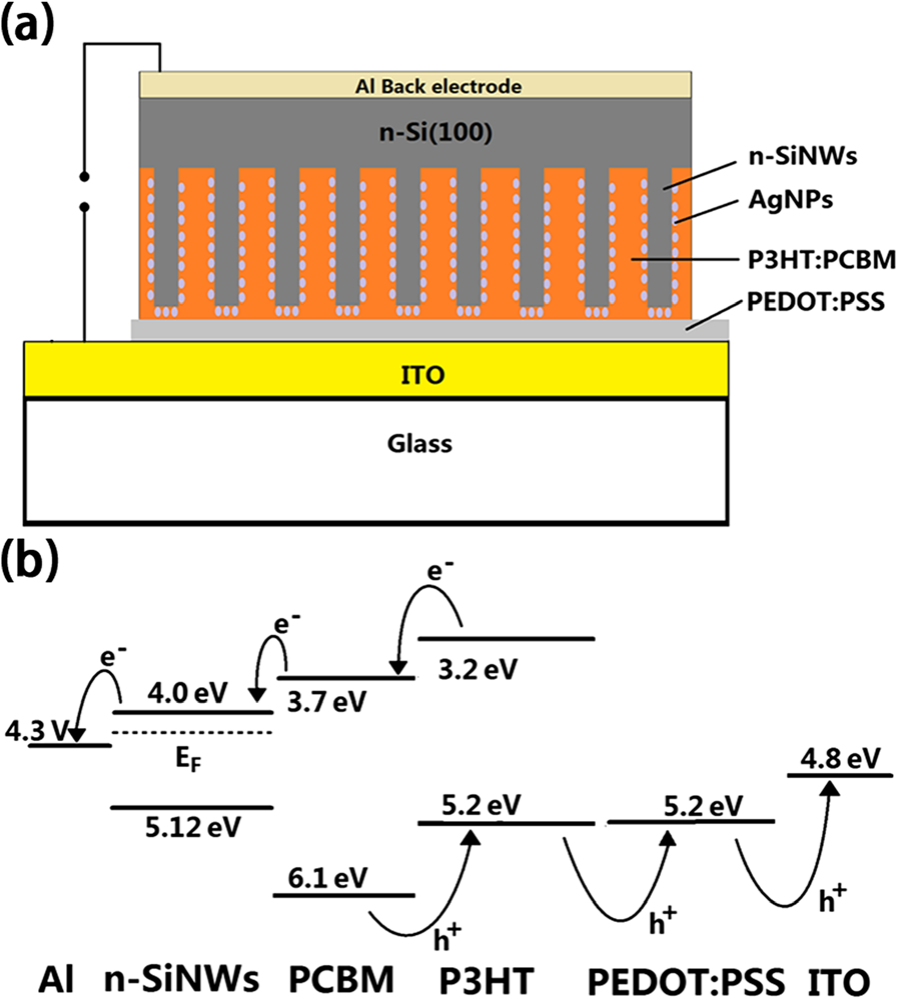
**Schematic of the AgNP-decorated SiNW/organic hybrid solar cell.** (**a**) Al/n-SiNW/PCBM:P3HT/PEDOT:PSS/ITO solar cell structure. (**b**) Energy band diagram and possible charge transportation of solar cell.

The schematic and energy band diagrams of our hybrid solar cells are shown in Figure 1. As shown, our hybrid cells could be treated as double-junction tandem solar cells [26,27]. The highest occupied molecular orbital of P3HT is positioned to inject holes into PEDOT:PSS and hence into the ITO electrode. The lowest unoccupied molecular orbital of PCBM is well above the Fermi level of the n-SiNWs, and electron collection should occur efficiently at the silicon interface. Electrons generated in the SiNWs will be collected at the Al electrode.

**Figure 2:**
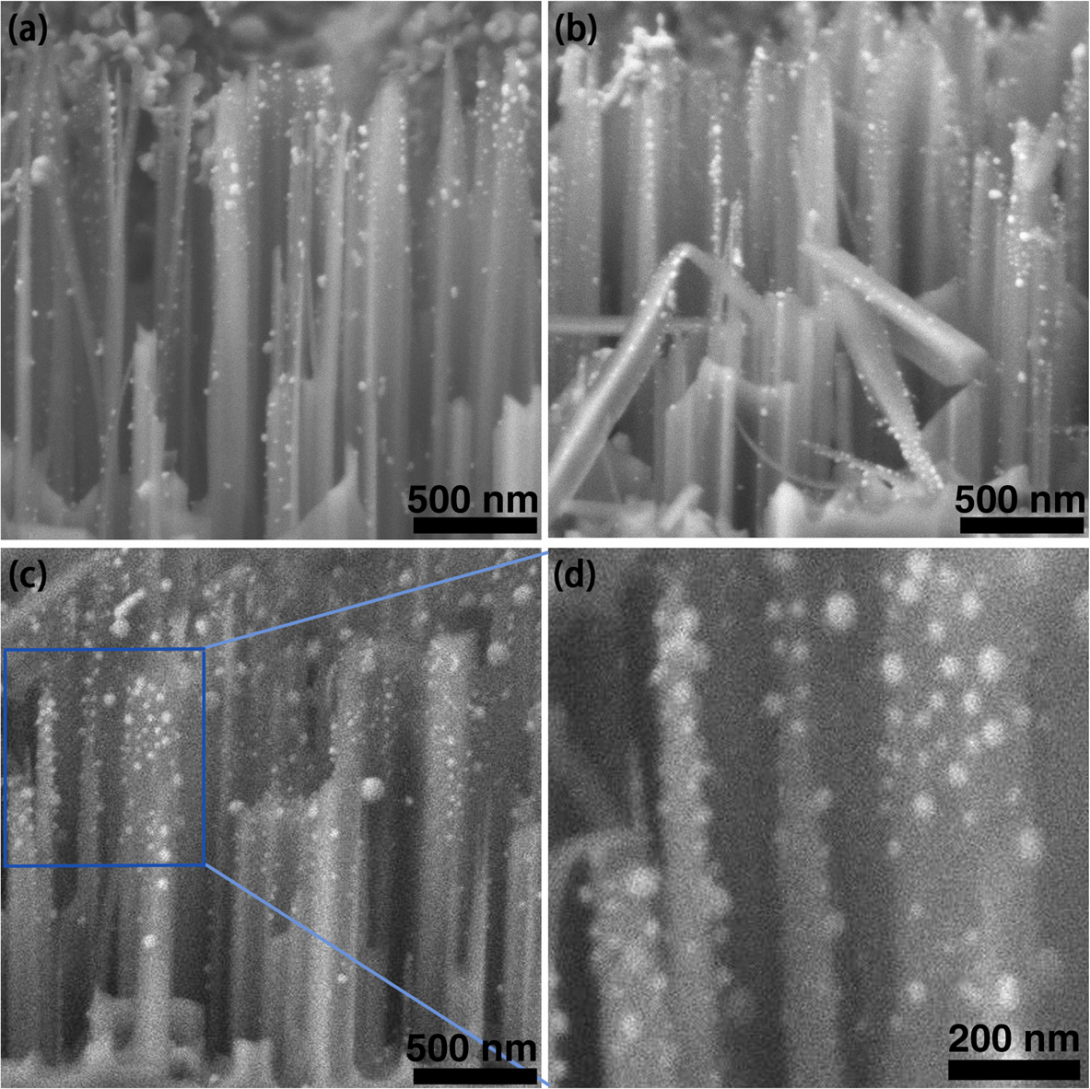
**SEM images of AgNP-decorated SiNW arrays.** (**a**, **b**, **c**) Side view of SiNW arrays after depositing AgNPs for (a) 4, (b) 6, and (c) 8 s. (**d**) A higher magnification image of AgNPs in (c).

Figure 2a,b,c shows the cross-sectional view of SiNW arrays after depositing AgNPs for 4, 6, and 8 s. It can be seen that the as-synthesized SiNWs are vertically aligned on the silicon surface. The average diameter and length of SiNWs are about 150 nm and 1.5 μm, respectively. The AgNPs prepared by deposition for 4, 6, and 8 s give average diameters of about 19, 23, and 26 nm, respectively. For longer deposition time, some Ag dendrite structures will form on top of the SiNW array; they will restrain the growth of AgNPs on the SiNW surface and worsen the spin coating effect in the post steps. So we only chose these three cases.

**Figure 3:**
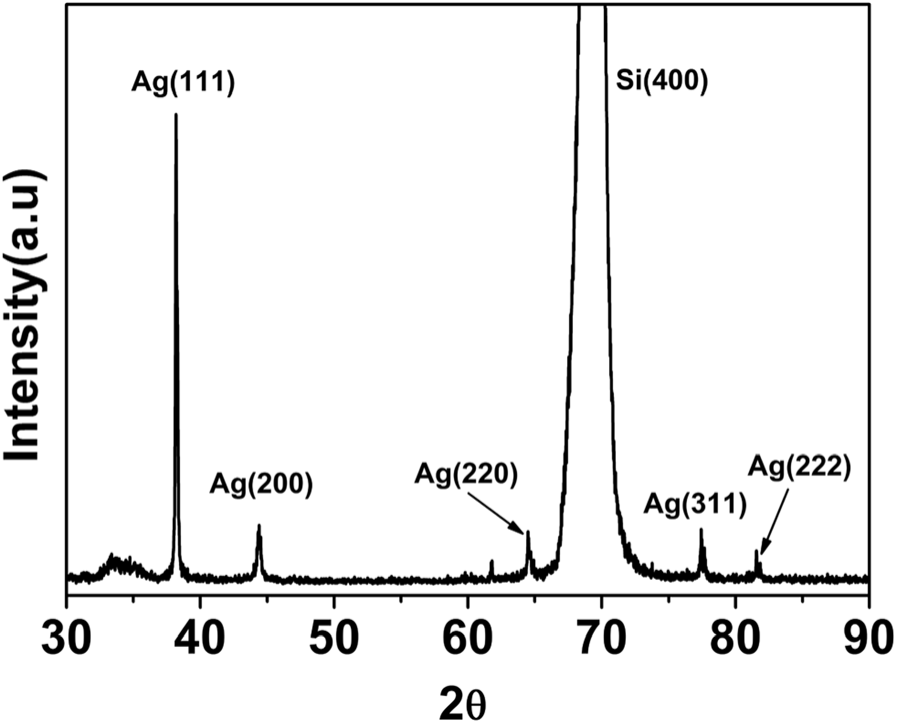
**XRD pattern of AgNPs on SiNW array.** Sample is obtained by depositing AgNPs for 8 s.

A typical closer look (Figure 2d) shows that AgNPs are well attached to the SiNW surface and predominantly spherical in shape after annealing treatment. Figure 3 shows the XRD pattern of AgNPs on SiNW array presented in Figure 2d. The sharp peaks that appeared in the XRD patterns can be assigned to Ag crystals, which illustrate good crystallinity of AgNPs after annealing treatment. However, one can see that the diameter of AgNPs actually ranges from 10 to 50 nm; this broad size distribution may lead to a possible optical response featured by multiple plasmon resonances.

**Figure 4:**
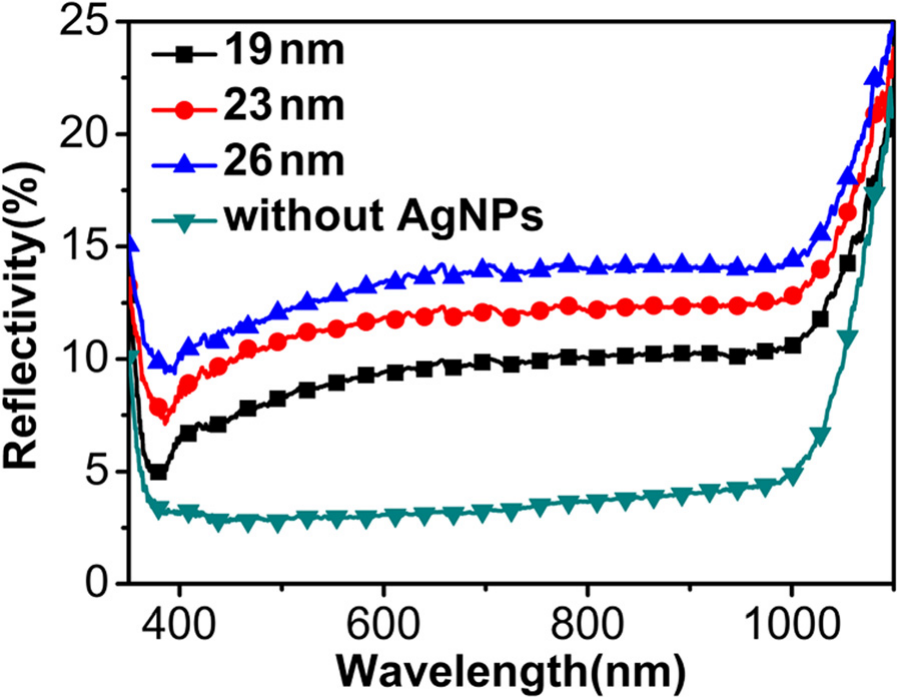
**Optical reflectance spectra of SiNW arrays.** The black square line, red dot line, and blue up-triangle line represent the spectra of SiNW arrays decorated with AgNPs with the diameter of 19, 23, and 26 nm, respectively. The green down-triangle line represents the reflectance of bare SiNW array without AgNPs.

Figure 4 shows the reflectivity of the SiNW array with and without AgNPs. Results of three different average diameters are presented to show the influence of particle size on light scattering effect. It clearly shows that the as-synthesized SiNWs on silicon substrate remarkably reduce reflectance throughout the entire wavelength range. This low reflectance of SiNWs mainly comes from the multiple reflection of light among SiNW array, which can lengthen the optical path and increase the capture ratio of photon. In AgNP-decorated cases, the reflectance curves lift up a little more than those in bare SiNW array, indicating the scattering effect of AgNPs. However, at the same time, it demonstrates a clear dip around 380 nm in the reflectance of AgNP-decorated samples, indicating the plasmon resonance absorption of the AgNPs. Furthermore, with the AgNP average size increasing from 19 to 26 nm, some particles become irregular in shape, which makes the resonance dip to broaden and show a red shift. Nevertheless, because the feature size of the particles is in the range of 19 to 26 nm, scattering behavior will be stronger than absorbing behavior on the whole.

It is well known that the transmittance of silicon in the wavelength region of 300 to 1,000 nm is almost zero [1]. Therefore, the absorbance of silicon will be directly related to the reflectance. It should also be noticed that the reflected light only contains the part of scattering light which escapes from the structure. Other scattering light from AgNPs will be absorbed by the adjacent SiNWs or experience multiple reflections in the structure. On the other hand, the scattering effect is relative to the dielectric around the particles. That is to say, only after incorporating the polymer into the space of the structure could the scattering light be utilized effectively.

**Figure 5:**
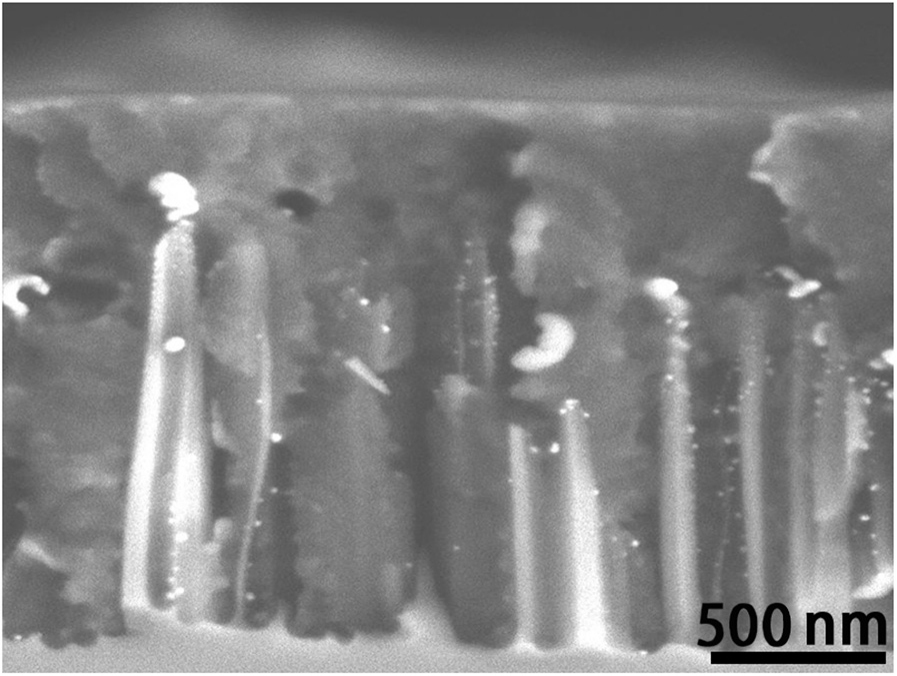
Cross-sectional morphology of SiNW array incorporated by P3HT/PCBM.

To make the SiNW and polymer composite together efficiently, we deposited polymer onto SiNWs by spin coating at a relative low rotation speed. Figure 5 shows the SEM image of the SiNW array incorporated by P3HT/PCBM. It can be seen that the polymer fills all the space among the SiNWs, which could make the polymer to wrap up all the SiNWs and AgNPs. This structure could provide many benefits for our solar cells. On the one hand, the SiNWs provide high-mobility pathways for carriers. On the other hand, uniformly distributed SiNWs, as supporters of AgNPs, ensure less agglomeration and good dispersity of AgNPs in the organic layer. In device manufacturing process, we directly coated a PEDOT:PSS/ITO/glass substrate on P3HT:PCBM to form a contact. Compared with sputtering, this method could reduce the structure damage of the polymer introduced by particle impact. At the same time, the glass substrate could prevent oxidation of the organic layer effectively, which is good for preserving the devices for a long time.

**Figure 6:**
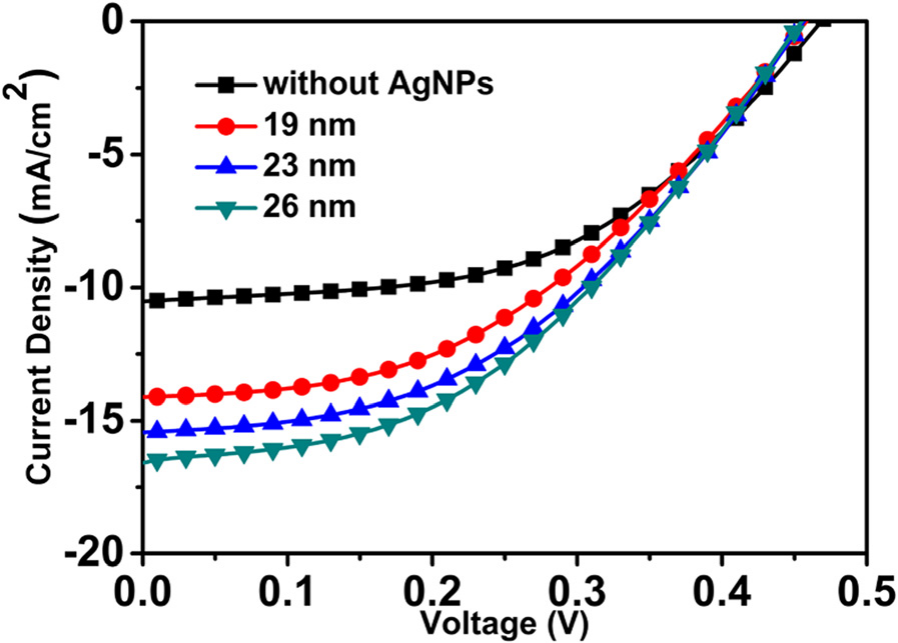
***J*-*V *characteristics of SiNW/organic hybrid solar cell.** The red dot line, blue up-triangle line, and green down-triangle line represent the *J*-*V* characteristics of SiNW arrays decorated with AgNPs with diameters of 19, 23, and 26 nm, respectively. The black square line represents the *J*-*V* characteristics of bare SiNW array without AgNPs.

The *J*-*V* characteristics of hybrid solar cells with different diameters of AgNPs compared to those of hybrid solar cells without AgNPs are shown in Figure 6. The short-circuit current density (*J*_sc_), open-circuit voltage (*V*_oc_), fill factor (FF), and efficiency (*η*) of all the cells are listed in Table 1. From the results presented in Figure 6 and Table 1, it can be found that the device performance of AgNP-decorated hybrid solar cells is improved compared to that of the reference device, which could be attributed to the enhanced light absorption of the polymer film. The short-circuit current increases from *J*_sc_ = 10.5 mA/cm^2^ for the reference cell to 16.6 mA/cm^2^ for the best AgNP-decorated cell, with an enhancement up to 58%. The current gain gives a rise of the conversion efficiency from *η* = 2.47% to 3.23%, whereas the fill factor reduces from 0.501 to 0.429. Within the group of AgNP-decorated cells, the diameter of the AgNPs is an important factor in determining the cell efficiency. As shown in the curves, as the AgNPs become bigger, the *J*_sc_ of the cell increases. This improvement of *J*_sc_ can be mainly attributed to the enhancement of light scattering as the AgNP diameter increases. That is to say, increased light scattering will lead to some increased lateral reflection of light among the SiNWs and absorption of light in the polymer. Higher absorption of light will introduce more photogenerated carriers and lead to improved current density [1,15].

**Table 1:** Device performances of SiNW/organic hybrid solar cells

**Device**	***J***_**sc **_**(mA/cm**^**2**^**)**	***V***_**oc **_**(V)**	**FF (%)**	***η *(%)**	***R***_**S **_**(Ω cm**^**2**^**)**
Without AgNPs	10.5	0.469	50.1	2.47	30.3
19 nm	14.1	0.458	43.4	2.81	26.8
23 nm	15.4	0.456	44.1	3.11	20.7
26 nm	16.6	0.455	42.9	3.23	19.8

However, we note that the *V*_oc_ of AgNP-decorated cells decreases lightly. It has been reported that the passivation provided by the polymer and the interface area between the polymer and SiNWs (or AgNPs) could influence the open-circuit voltage of the devices [1]. In other words, increased AgNP diameter will lead to some increased interface area and hence decreased *V*_oc_. It should be mentioned that the fill factor of all the hybrid cells are still very low. The series resistance comes from defects in the SiNW array, and poor electrode contact might be responsible for the low value.

**Figure 7:**
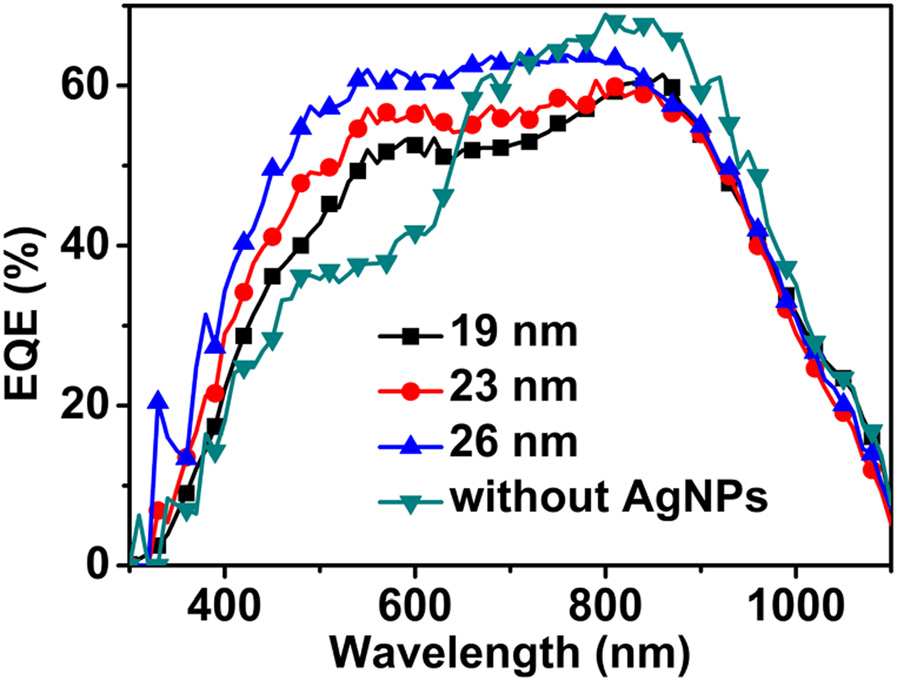
**EQE spectra of SiNW/organic hybrid solar cell.** The black square line, red dot line, and blue up-triangle line represent the EQE of SiNW arrays decorated with AgNPs with diameters of 19, 23, and 26 nm, respectively. The green down-triangle line represents the EQE of bare SiNW array without AgNPs.

External quantum efficiency (EQE) measurements of the cells with and without AgNPs have been carried out for comparison, as shown in Figure 7. Since silicon substrate and P3HT/PCBM have different absorption spectra and our devices could be treated as double-junction tandem solar cells, the results of wavelength-dependent response of cells will reflect the light scattering effect of AgNPs more visually. It could be seen that the presence of the AgNPs leads to a considerable improvement in EQE for short-wavelength range, which is consistent with the absorption spectra of P3HT:PCBM [23], as compared to the reference cells. Furthermore, the curves of AgNP-decorated cells decrease slightly in long-wavelength range. This decrease could be attributed to the low light absorption in the silicon layer reduced by scattering and low absorptivity of the polymer in this wavelength range. However, it seems that there is no obvious difference of EQE among AgNP-decorated samples in the wavelength region of 800 to 1,000 nm. This phenomenon might be closely related to the optical confinement effect in the long-wavelength region. It has been reported that a dielectric shell surrounding SiNWs significantly reinforced their optical confinement and caused their resonant wavelength to red shift [28,29]. In our hybrid structure, the P3HT:PCBM layer surrounding SiNWs could also induce a similar optical confinement. This effect resulted in considerable improvement in light absorption of low-energy photons, which could diminish the difference of reflectance among AgNP-decorated samples in the wavelength region of 800 to 1,000 nm.

Although the efficiencies of our devices are much lower than those of commercial silicon solar cells, the results of our experiments proved good effects of AgNPs in the SiNW/organic hybrid solar cell very well. Several other methods may be used to increase the efficiency of this hybrid solar cell. For example, etching the silicon substrate with an anodic aluminum oxide template could obtain a SiNW array with controlled size and excellent uniform distribution [30]. If we used a small-sized SiNW array to manufacture hybrid solar cells, the organic layer would become thinner, resulting in the improvement of carrier collection efficiency. On the other hand, a gas-phase polymerization method could be introduced in the polymer coating process to form a uniform thin layer on SiNWs, resulting in a core-shell-structured solar cell with lateral heterojunction [31]. Therefore, further efforts should be focused on these issues to improve the properties of SiNW/organic hybrid solar cells.

## Conclusions

In summary, AgNP-decorated SiNWs were fabricated by metal-assisted chemical etching and electroless deposition. AgNP-decorated SiNW/organic hybrid solar cells were also demonstrated, treating them as double-junction tandem solar cells. The power performance of cells is enhanced by using AgNPs. The performance is dominated by current enhancement. The short-circuit current increases from *J*_sc_ = 10.5 mA/cm^2^ for the reference cell to 16.6 mA/cm^2^ for the best AgNP-decorated cell, with an enhancement up to 58%. The current gain gives a rise of the conversion efficiency from *η* = 2.47% to 3.23%, with an enhancement up to 30%. This enhancement is explained by light trapping effect of SiNWs and surface plasmon resonance scattering of AgNPs.

## Competing interests

The authors declare that they have no competing interests.

## Authors' contributions

KL participated in the design of the study, carried out the total experiment, performed the statistical analysis, as well as drafted the manuscript. SQ participated in the guidance of the experiment. XZ helped give the corrections of the manuscript. ZW helped give the theoretical guidance of the experiment. FT gave some help in obtaining the reading papers. All authors read and approved the final manuscript.
